# Phytochemical Study of *Euphorbia turcomanica* Boiss.

**DOI:** 10.3390/metabo12121200

**Published:** 2022-11-30

**Authors:** Newsha Motinia, Mustafa Ghannadian, Behzad Zolfaghari, Zeinab Yazdiniapour

**Affiliations:** Department of Pharmacognosy, Isfahan University of Medical Sciences, Isfahan 81746-73461, Iran

**Keywords:** *Euphorbia turcomanica*, phytochemistry, terpenoid, triterpene, steroid

## Abstract

The attraction to the *Euphorbia* genus, with its remarkable diversity in species, is due to its variety of chemical compositions. *Euphorbia turcomanica* is one of the species of the spurge family growing wildly in Iran. This research aims to investigate the presence of secondary metabolites, specially terpenoid compounds, in terms of structural determination. Samples of *E. tucomanica* were finely powdered and macerated with acetone/dichloromethane 2:1. Repeated column chromatography using silica gel, MPLC, and HPLC methods followed by the analysis of data obtained from spectroscopic means was carried out to purify and identify the terpenoid constituents. The chemical structures of nine known terpenoids were determined for the first time from *E. turcomanica* during this study. Loliolide (**1**), a monoterpene, and eight steroids and triterpenes, including simiarenol (**2**), isomultiflorenol (**3**), cycloart-25-ene-3β,24-diol (**4**), β-sitosterol (**5**), cycloart-23-ene-3β,25-diol (**6**), 3α, 11α-dihydroxyurs-12-ene (**7**), 3β, 24β, 25-trihydroxycycloartane (**8**), and 7α-hydroxystigmasterol (**9**) were isolated and identified. *E. tucomanica,* with a rich terpenoid profile, can be one of the valuable and economic sources providing compounds for drug development.

## 1. Introduction

The family Euphorbiaceae, with nearly around 7500 species and 300 genera, is one of the largest families of flowering plants on earth. More than 2000 species belong to the genus *Euphorbia* or spurge, which is the largest genus of this family. These plants occur naturally in both tropical and temperate regions [1,2]. Iran, with 92 species of *Euphorbia*, is one of the most diverse countries in southwest Asia, with the highest occurrence of endemics which are 21 species until 2020 [3]. *Euphorbia turcomanica* Boiss., one of the species of the spurge family, is an annual herb growing wild in the lower fields and plains of Iran [4].

Secondary metabolites with a wide range of biological activities and broad structural diversity have been isolated from different parts of *Euphorbia* species, including bark, cortex, seeds, latex, lactiferous duct, stem, wood, stem bark, leaves, and flowers. The complex and diverse phytochemical properties of this genus include compounds from different chemical classes, especially the ones that consist of isoprene units known as terpenes and terpenoids, which are considered the largest and most diverse group of natural products. Between the major classes of terpenoids found in *Euphorbia* species, diterpenes and triterpenes are the most important constituents of the secondary metabolites, mainly in their ester forms [5,6]. Diterpenes are prominent constituents isolated from this genus incorporating more than 350 new diterpenes over the last decade with over 20 different skeletal structures. Macrocyclic diterpenes are the most frequent diterpenoids from *Euphorbia* plants. Some of these diterpenoids are scarcely distributed in nature, like tigliane, which can be found in *Euphorbia* species [7,8]. The second most extensive group of chemical constituents in this genus are triterpenes, with more than 130 different triterpenoids varying in 10 different structural forms with a significant diversity amongst the cyclic triterpenes. The tetracyclic and pentacyclic triterpenoids and steroids are isolated triterpenes from many species in this genus. Euphane and tirucallane triterpenoids are not only exclusively found in this genus but occur most frequently in them, which is a distinguished point about members of the Euphorbiaceae family and consequently can be considered a valuable source for these compounds [2,9].

In traditional medicine, species of *Euphorbia* have a wide range of applications, but in particular, the latex of *E. turcomanica* is used as a laxative and diuretic. Also, it is externally applied to snake bites and scorpion stings and internally used for treating intestinal worms and purifying blood [10]. The attraction to the *Euphorbia* genus in pharmacological research is due to the variety of chemical compositions in them. Part of these effects is associated with the presence of terpenoids, including diterpenes and triterpenes, especially cycloartanes. Plants-derived terpenoids have been identified to have anticancer and anti-inflammatory activities. The *Euphorbia* genus, with a rich terpenoid profile, can be one of the valuable and economically important sources providing compounds in drug development [9,11].

Most research has proven the biological and therapeutic properties of *E. turcomanica,* but only a few phytochemical studies have been carried out to isolate and elucidate the chemical constituents present in this plant, including steroids, flavonoids, phenolics, aromatics, and especially terpenoid compounds, by preliminary screening [12].

This study describes the isolation and structural determination of secondary metabolites from *E. turcomanica*. The structures of these compounds were mainly established by spectroscopic methods.

## 2. Materials and Methods

### 2.1. General Experimental Procedures

Medium pressure liquid chromatography (MPLC) was carried out on a BUCHI^®^ Gradient SystemC605 apparatus equipped with a fraction collector, using a glass column filled with silica gel (particle size 15–40 µm; Merck^®,^ Darmstadt, Germany), HPLC (High-Performance Liquid Chromatography) on a Waters^®^ apparatus equipped with a 515 pump, a Waters^®^2487 Dual Wavelength Absorbance Detector and a Waters^®^2414 Refractive Index Detector, using Spherisorb^®^Sil (250 mm × 20 mm) column, 5 µm silica gel and hexane, with ethyl acetate as the mobile phase. The purity was checked using TLC (Thin-Layer Chromatography) with Merck^®^ silica gel GF254 plates detected by spraying cerium sulfate. Determination of each structure was performed by NMR spectra recorded on a Bruker Avance^®^ AV400 spectrometer.

### 2.2. Plant Materials

The whole plant of *E. turcomanica* was gathered in two places, Isfahan-Qom Road, Center of Iran, and Bandar Tourkaman, North of Iran, during the flowering time in July 2012. The plant was identified by Mr. Bahram Zehzad, Department of biology, University of Shahid Beheshti, Tehran, and a voucher specimen (No. 2410) is kept at the Herbarium of Pharmacognosy, Department of Pharmacognosy, Faculty of Pharmacy, Isfahan University of Medical Sciences.

### 2.3. Extraction Procedure

Air-dried samples of *E. tucomanica* were finely powdered and extracted with acetone/dichloromethane 2:1. The maceration was carried out at room temperature in 3 repeated procedures that each took 4 days and 10–15 liters of solvent. The extract was concentrated under a vacuum. The greasy extract was subjected to Celite and chromatographed using the VLC method with silica gel coated with paraffin as the stationary phase and MeOH–H_2_O (60:40) and MeOH–H_2_O (90:10) as a solvent system to eliminate chlorophyll and fats from the extract. Normal-phase (NP)-TLC analysis indicated that the fraction which was separated by MeOH–H_2_O (60:40) contained a series of terpenoids and fraction eluted with MeOH–H_2_O (90:10) affording steroid and triterpene-containing extract.

### 2.4. Isolation of Terpenoids

The terpene-containing extract was dissolved in CHCl_3_, adsorbed onto Celite, and chromatographed using an open column and a linear gradient solvent system from n-hexane/ EtOAc 3–30%, which yielded 15 fractions. After comparing the TLC analysis of the obtained fractions, the ones with similar UV characterizations and Rf values were combined and concentrated by vacuum. Further purification of these fractions was carried out by MPLC using a linear gradient solvent system from MeOH/H_2_O (70:30) to MeOH/H_2_O (100:0), resulting in subfractions [13]. Compound **1** was obtained from open column chromatography using a linear gradient solvent system from n-hexane/acetone 3% to n-hexane/acetone 30% of the first subfraction of fraction 10 in an n-hexane/acetone 20% mobile phase. After further vacuum concentration and analysis, it was identified as pure crystals of loliolide.

The steroid and triterpene-containing extract chromatographed by MPLC using a linear gradient solvent system from n-hexane 100% to EtOAc 100% [13]. Fractions were analyzed by TLC, and similar fractions were mixed and concentrated under a vacuum. Seventeen fractions were obtained and subjected to TLC and preliminary NMR analysis.

Fraction 3 was eluted with Hex/EtOAc (90:10) in MPLC and further purified by HPLC using Hex/EtOAc (90:10) as the mobile phase and resulting in the isolation of compound **2** as simiarenol and [3] as isomultiflorenol. Fractions 5 and 6 were both eluted by Hex/EtOAc (70:30) and applied on HPLC using Hex/EtOAc (80:20) as solvent. Compound **4** was isolated from fraction 5 as cycloartane. Compound **5** was purified from obtained crystals by recrystallization and identified as pure β-sitosterol. Fractions 7 and 8 were eluted with Hex/EtOAc (70:30) in MPLC and purified by HPLC using Hex/EtOAc (80:20) as a solvent and resulting in the isolation of compound **6** as a cycloartane. Fraction 8 contained pure crystals that were filtered and concentrated by a rotary evaporator, which were further purified by recrystallization and yielded compound **7**. Fraction 12, eluted with Hex/EtOAc (50:50), was purified further on HPLC using Hex/EtOAc (37:63) as a solvent to yield compound **8** as a cycloartane-type triterpene. Fraction 14 was eluted with Hex/EtOAc (40:60) in MPLC and further purified by HPLC using Hex/EtOAc (50:50) as solvent. As a result, a significant amount of needle-shaped crystal was obtained and further purified by recrystallization and yielded compound **9** as a steroidal triterpene.

## 3. Results

### 3.1. Spectral Data of Compounds **1**–**9**

**Figure 1:**
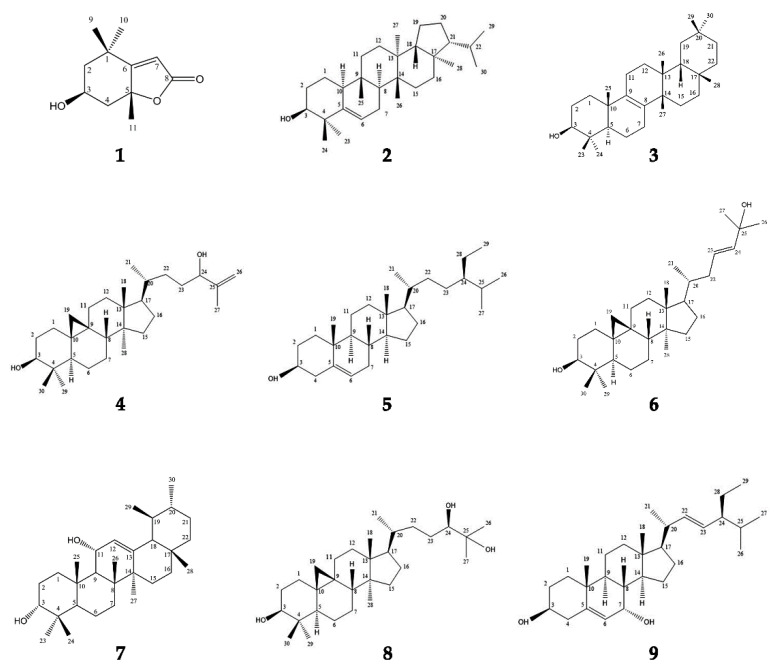
Chemical structures of compounds **1–9**.

Nine compounds were extracted and purified from *Euphorbia turcomanica,* as shown in Figure 1. They were identified using spectroscopic analysis. The ^1^H NMR and ^13^C NMR data of each compound are reported here.

1. Loliolide (Compound **1**)

White crystal, MW (g/mol): 196.1; ^1^H NMR data (CDCI_3_, 400 MHz): δ_H_ 1.55 (dd, J = 14.8-3.6 Hz, H-2a), 1.98 (t, J = 2.4 Hz, H-2b), 4.35 (m, H-3), 1.80 (dd, J = 13.2-4.0 Hz, H-4a), 2.02 (t, J = 2.8 Hz, H-4b), 5.72 (m, H-7), 1.49 (s, H-9), 1.29 (s, H-10), 1.80 (d, J = 0.8 Hz, H-11), ^13^C NMR data (CDCl_3_, 100 MHz): 30.7 (C-1), 45.6 (C-2), 66.8 (C-3), 47.3 (C-4), 86.7 (C-5), 171.9 (C-6), 112.9 (C-7), 182.4 (C-8), 26.5 (C-9), 27.0 (C-10), 30.7 (C-11).

2. Simiarenol: 3β-hydroxy-E:B-friedo-hop-5-ene (Compound **2**)

Amorphous white powder, MW (g/mol): 426.70; ^1^H NMR data (CDCI_3_, 400 MHz): δ_H_ 3.47 (dd, J = 10.9-5.7 Hz, H-3a), 5.62 (dd, J = 6.0-6.0 Hz, H-6), 1.35 (m, H-22), 1.25 (s, H-23), 1.14 (s, H-24), 1.04 (s, H-25), 1.00 (s, H-26), 0.92 (s, H-27), 0.77 (s, H-28), 0.84 (d, J = 6.5 Hz, H-29), 0.88 (d, J = 6.5 Hz, H-30), ^13^C NMR data (CDCl_3_, 100 MHz): 18.20 (C-1), 27.90 (C-2), 76.52 (C-3), 40.97 (C-4), 142.08 (C-5), 122.17 (C-6), 24.18 (C-7), 44.37 (C-8), 34.97 (C-9), 50.35 (C-10), 34.27 (C-11), 29.21 (C-12), 38.74 (C-13), 39.09 (C-14), 29.08 (C-15), 35.53 (C-16), 42.92 (C-17), 51.86 (C-18), 20.05 (C-19), 28.46 (C-20), 60.15 (C-21), 30.94 (C-22), 29.11 (C-23), 25.62 (C-24), 18.01 (C-25), 15.89 (C-26), 15.14 (C-27), 16.21 (C-28), 22.10 (C-29), 23.07 (C-30).

3. Isomultiflorenol (Compound **3**)

Amorphous white powder, MW (g/mol): 426.7; ^1^H NMR data (CDCI_3_, 400 MHz): δ_H_ 3.23 (dd, J = 11.6-4.6 Hz, H-3a), 0.80 (s, H-23), 0.95 (s, H-24), 0.95 (s, H-25), 1.05 (s, H-26), 0.96 (s, H-27), 1.06 (s, H-28), 0.98 (s, H-29), 1.00 (s, H-30), ^13^C NMR data (CDCl_3_, 100 MHz): 34.33 (C-1), 26.34 (C-2), 79.17 (C-3), 38.94 (C-4), 50.81 (C-5), 19.36 (C-6), 28.04 (C-7), 135.19 (C-8), 133.63 (C-9), 37.72 (C-10), 20.95 (C-11), 30.80 (C-12), 37.39 (C-13), 41.13 (C-14), 27.63 (C-15), 36.89 (C-16), 30.93 (C-17), 44.23 (C-18), 34.48 (C-19), 28.47 (C-20), 35.15 (C-21), 36.84 (C-22), 28.18 (C-23), 15.76 (C-24), 19.96 (C-25), 19.14 (C-26), 24.78 (C-27), 31.65 (C-28), 34.74 (C-29), 33.09 (C-30).

4. Cycloart-25-ene-3β,24-diol (Compound **4**)

Amorphous white powder, MW (g/mol): 442.38; ^1^H NMR data (CDCI_3_, 400 MHz): δ_H_ 3.28 (dd, J = 11.0-4.5 Hz, H-3a), 0.96 (s, H-18), 0.33 (d, J = 4.0 Hz, H-19a), 0.55 (d, J = 4.0 Hz, H-19b), 0.88 (d, J = 6.4 Hz, H-21), 4.02 (t, J = 6.03 Hz, H-24), 1.72 (s, H-27), 0.88 (s, H-28), 0.80 (s, H-29), 0.96 (s, H-30), ^13^C NMR data (CDCl_3_, 100 MHz): 32.04 (C-1), 30.49 (C-2), 78.99 (C-3), 40.61 (C-4), 47.21 (C-5), 21.25 (C-6), 28.22 (C-7), 48.14 (C-8), 20.09 (C-9), 26.48 (C-10), 26.15 (C-11), 35.67 (C-12), 45.38 (C-13), 48.93 (C-14), 32.08 (C-15), 26.56 (C-16), 52.27 (C-17), 18.19 (C-18), 29.85 (C-19), 36.05 (C-20), 18.44 (C-21), 32.10 (C-22), 28.28 (C-23), 76.52 (C-24), 147.87 (C-25), 111.10 (C-26), 17.74 (C-27), 19.45 (C-28), 25.56 (C-29), 14.15 (C-30).

5. β-sitosterol: Stigmast-5-en-3β-ol (Compound **5**)

White crystal, MW (g/mol): 414; ^1^H NMR data (CDCI3, 400 MHz): δ_H_ 1.85 (m, H-1), 3.52 (m, H-3a), 5.36 (br s, H-6), 0.68 (s, H-18), 1.01 (s, H-19), 0.92 (d, J = 6.4 Hz, H-21), 0.81 (d, J = 6.9 Hz, H-26), 0.85 (d, J = 7.7 Hz, H-27), 0.86 (t, J = 7.5 Hz, H-29), 0.96 (s, H-30), ^13^C NMR data (CDCl_3_, 100 MHz): 37.37 (C-1), 29.23 (C-2), 71.95 (C-3), 42.42 (C-4), 140.88 (C-5), 121.88 (C-6), 32.02 (C-7), 32.04 (C-8), 50.23 (C-9), 36.63 (C-10), 21.21 (C-11), 39.89 (C-12), 42.44 (C-13), 56.88 (C-14), 24.44 (C-15), 28.40 (C-16), 56.15 (C-17), 12.00 (C-18), 19.55 (C-19), 36.28 (C-20), 18.91 (C-21), 34.05 (C-22), 26.13 (C-23), 45.93 (C-24), 29.23 (C-25), 19.98 (C-26), 19.15 (C-27), 23.17 (C-28), 12.12 (C-29).

6. Cycloart-23-ene-3β,25-diol (Compound **6**)

White crystal, MW (g/mol): 442.38; ^1^H NMR data (CDCI_3_, 400 MHz): δ_H_ 3.28 (dd, J = 11.0-4.5 Hz, H-3a), 0.97 (s, H-18), 0.33 (d, J = 4.3 Hz, H-19a), 0.55 (d, J = 4.4 Hz, H-19b), 0.86 (d, J = 6.4 Hz, H-21), 5.60 (m, H-23), 5.60 (m, H-24), 1.32 (s, H-26), 1.32 (s, H-27), 0.88 (s, H-28), 0.81 (s, H-29), 0.97 (s, H-30), ^13^C NMR data (CDCl_3_, 100 MHz): 32.08 (C-1), 30.49 (C-2), 78.98 (C-3), 40.61 (C-4), 47.20 (C-5), 21.25 (C-6), 28.21 (C-7), 48.12 (C-8), 20.09 (C-9), 26.14 (C-10), 25.57 (C-11), 32.88 (C-12), 45.41 (C-13), 48.94 (C-14), 35.70 (C-15), 26.54 (C-16), 52.12 (C-17), 18.24 (C-18), 30.00 (C-19), 36.51 (C-20), 18.41 (C-21), 39.16 (C-22), 139.42 (C-23), 125.76 (C-24), 70.73 (C-25), 30.10 (C-26), 30.04 (C-27), 19.43 (C-28), 14.15 (C-29), 24.85 (C-30).

7. 3α, 11α-dihydroxyurs-12-ene (Compound **7**)

White crystal, MW (g/mol): 442.38; ^1^H NMR data (CDCI_3_, 400 MHz): 3.21 (m, H-3b), 3.98 (m, H-11), 4.90 (d, J = 3.2Hz, H-12), 0.90 (s, H-23), 0.69 (s, H-24), 0.94 (s, H-25), 0.76 (d, J = 5.4 Hz, H-26), 1.00 (s, H-27), 0.82 (m, H-30), ^13^C NMR data (CDCl_3_, 100 MHz): 33.5 (C-1), 25.4 (C-2), 76.0 (C-3), 37.5 (C-4), 48.8 (C-5), 18.2 (C-6), 35.2 (C-7), 43.5 (C-8), 55.8 (C-9), 38.2 (C-10), 68.4 (C-11), 128.7 (C-12), 142.9 (C-13), 42.2 (C-14), 27.9 (C-15), 27.7 (C-16), 33.6 (C-17), 58.1 (C-18), 39.4 (C-19), 39.3 (C-20), 31.1 (C-21), 41.3 (C-22), 28.7 (C-23), 22.4 (C-24), 16.6 (C-25), 18.0 (C-26), 23.3 (C-27), 28.6 (C-28), 17.5 (C-29), 21.3 (C-30).

8. 3β, 24β, 25-trihydroxycycloartane (Compound **8**)

Amorphous white powder, MW (g/mol): 460.39; ^1^H NMR data (CDCI_3_, 400 MHz): δ_H_ 3.27 (m, H-3a), 0.96 (s, H-18), 0.33 (d, J = 4.2 Hz, H-19a), 0.55 (d, J = 4.2 Hz, H-19b), 0.88 (d, J = 5.2 Hz, H-21), 3.31 (m, H-24), 1.16 (s, H-26), 1.22 (s, H-27), 0.89 (s, H-28), 0.96 (s, H-29), 0.80 (s, H-30), ^13^C NMR data (CDCl_3_, 100 MHz): 32.10 (C-1), 30.49 (C-2), 78.99 (C-3), 40.62 (C-4), 47.23 (C-5), 21.26 (C-6), 28.54 (C-7), 48.14 (C-8), 20.10 (C-9), 26.58 (C-10), 26.15 (C-11), 35.68 (C-12), 45.42 (C-13), 50.59 (C-14), 33.03 (C-15), 26.68 (C-16), 52.44 (C-17), 18.22 (C-18), 30.05 (C-19), 36.52 (C-20), 18.57 (C-21), 33.66 (C-22), 28.68 (C-23), 79.77 (C-24), 73.36 (C-25), 23.36 (C-26), 26.72 (C-27), 19.44 (C-28), 14.15 (C-29), 25.57 (C-30).

9. 7α-hydroxystigmasterol (Compound **9**)

Needle white crystal, MW (g/mol): 428.70; ^1^H NMR data (CDCI_3_, 400 MHz): δ_H_ 3.58 (m, H-3a), 2.33 (dd, J = 5.2-1.8 Hz, H-4), 2.28 (t, J = 2.0 Hz, H-5), 5.64 (dd, J = 5.4-1.9 Hz, H-6), 3.85 (br s, H-7), 0.68 (s, H-18), 0.99 (s, H-19), 0.93 (d, J = 6.5 Hz, H-21), 5.16 (dd, J = 15.2-8.6 Hz, H-22), 0.81 (d, J = 6.9 Hz, H-26), 0.83 (d, J = 6.9 Hz, H-27), 0.83 (t, J = 7.3 Hz H-29), ^13^C NMR data (CDCl_3_, 100 MHz): 37.13 (C-1), 31.50 (C-2), 71.49 (C-3), 42.14 (C-4), 146.39 (C-5), 124.00 (C-6), 65.51 (C-7), 37.64 (C-8), 42.38 (C-9), 36.24 (C-10), 20.84 (C-11), 39.29 (C-12), 42.27 (C-13), 49.55 (C-14), 24.45 (C-15), 28.43 (C-16), 55.82 (C-17), 11.78 (C-18), 18.39 (C-19), 39.92 (C-20), 19.15 (C-21), 138.39 (C-22), 128.82 (C-23), 51.36 (C-24), 32.03 (C-25), 18.94 (C-26), 19.95 (C-27), 25.99 (C-28), 12.13 (C-29).

### 3.2. Structure Identification of Compounds **1**–**9**

Compound (**1**) was identified as C_11_H_16_O_3_. Based on ^1^H NMR, two methylic protons at δ_H_ 1.49 (H-9) and 1.29 (H-10) and one olefinic proton at δ_H_ 5.72 (H-7) were detected. The ^13^C NMR spectral data demonstrates the existence of a carbonyl group on the lactone ring (δc 182.4), a secondary hydroxyl group (δ_C_ 66.8), a trisubstituted olefinic bond (δ_C_ 112.9 and 171.9), and three singlet methyl groups (δ_C_ 26.5, 27.0, and 30.7) which has resemblance with the data from the literature. This structure was identified as loliolide [14,15].

Compound **2** was found to be C_30_H_50_O. The ^1^H NMR spectrum shows the existence of six tertiary methyl groups. The presence of the isopropyl group at C21 is confirmed by doublet signals at δ_H_ 0.84 and 0.88, which indicate two secondary methyls. Also, the δ_H_. 5.62 signal in the ^1^H NMR spectrum refers to the olefinic proton at H-6. The ^1^H NMR and ^13^C NMR spectra signals at δ_H_ 3.47 and δ_C_ 76.52 suggest that an oxymethine on C3 confirms the hydroxyl group α-orientation. Regarding these data and the findings from the published literature [16,17], compound **2** was identified as simiarenol.

Compound **3** with the molecular formula of C_30_H_50_O was elucidated as isomultiflorenol based on the spectral data analysis and comparing them to the literature [18]. The ^1^H NMR signals at δ_H_ 0.80 (H-23), 0.95 (H-24), 1.05 (H-25), 0.96 (H-27), 1.06 (H-28), 0.98 (H-29), and 1.00 (H-30) revealed eight tertiary methyl groups, and the double doublet signal at δ_H_ 3.23 (H-3) refers to the axially oriented hydroxyl group. Based on the ^13^C NMR and DEPT data, eight CH3 carbons, eleven CH2 carbons, and eight quaternary carbons are revealed. The ^13^C NMR spectrum also indicates the presence of the double bond correlated to the olefinic carbons at δ_C_ 135.19 (C-8) and 133.63 (C-9).

Compound **4** was isolated with the molecular formula of C_30_H_50_O_2_ and identified as cycloart-25-en-3β,24-diol according to its ^1^H NMR and ^13^C NMR data, compared with published data [19]. The ^1^H- NMR signals in the up-field area (δ_H_ 0.33 and 0.55) revealed a pair of doublets, which is characteristic of a cyclopropane ring. Also, it corroborated one secondary methyl group at 0.88 and five singlet methyls at δ_H_ 1.72 (H-27), 0.96 (H-18, H-30), 0.88 (H-28), and 0.80 (H-29). A double doublet carbinolic proton axially orientated at δ_H_ 3.28, assigned to the hydroxyl group as 3β-OH, is also determined from the ^1^H NMR data analysis. 

Compound **5** was found to be C_29_H_50_O, known as β-sitosterol. The ^1^H NMR data confirm the resemblance to Δ5-3β-hydroxy sterols by the presence of multiplets at δ_H_ 3.52, a characteristic peak for H-3α, and δ_H_ 5.36, determining the presence of the olefinic proton. The ^1^H NMR peaks at δ_H_ 1.01 (H-19) and 0.68 (H-18) reveal two singlet methyls, and the ones at δ_H_ 0.92 (H-21), 0.81 (H-26), and 0.92 (H-27) shows three secondary methyls and one methyl with a triplet signal at δ_H_ 0.86 (H-29). The ^13^C NMR spectrum shows two signals correlated with the olefinic carbons at δ_C_ 140.88 (C-5) and 121.88 (C-6), revealing the presence of the double bond. The signal at δ_C_ 71.95 suggests an oxymethine on C3, and the ones at δ_C_ 12.00 and 19.55 confirm the two methyl groups present at C18 and C19. The published spectroscopic data for β-sitosterol are all in agreement with these findings [20,21,22].

Compound **6** showed the molecular formula of C_30_H_50_O_2_ and was determined as cycloart-23-ene-3β,25-diol by comparison of the spectral data to those in the published literature [23,24,25]. Based on ^1^H NMR spectra, one oxymethine proton at δ_H_ 3.28 (H-3), five singlet methyls at 1.72 (H-27), 0.88 (H-28), 0.80 (H-29), 0.96 (H-18, H-30) and one further secondary doublet methyl at 0.88 (H-21) were detected. The pair of doublet signals at δ_H_ 0.55 and 0.33 in the up-field area are related to the cyclopropane ring in this structure.

Compound **7** assigned the molecular formula of C_30_H_50_O_2_ and, based on the comparison with those reported in the literature, was determined to be 3α, 11α- dihydroxyurs-12-ene [26]. The ^1^H NMR spectrum singly supports the existence of two hydroxyl groups at δ_H_ 3.21 (H-3) and 3.98 (H-11). The peak at δ_H_ 3.21 (H-3) and the ^13^C NMR peak at δ_C_ 76.0 (C-3) revealed the presence of a 3α-hydroxyl group that was supported by the coupling constants. It also explains the strengthened α-effect at δ_C_ 76.0 (C-3) and the diminished β-effect at δ_C_ 25.4 (C-2) and 37.3 (C-4), comparing to the data from structures with axial orientation. Analyzing the ^13^C NMR spectra suggested the presence of a urs-12-ene by the olefinic carbons at δ_C_ 128.7 and 142.9. The olefinic signal at δ_H_ 4.90 from ^1^H NMR was assigned to the H-12. The urs-12-ene skeleton is determined from the ^1^H NMR signals of eight methyl groups, two of which are on a methine group and six are on quaternary carbons. 

Compound **8** showed the molecular formula of C_30_H_52_O_3,_ and, based on the data and comparison of the spectral values with the literature, it was elucidated as 3β, 24β, and 25-trihydroxycycloartane [27]. The ^1^H NMR peaks at δ_H_ 1.16 (H-26), 1.22 (H-27), 0.89 (H-28), 0.96 (H-18, H-29), and 0.80 (H-30) revealed six tertiary methyl groups and the peak at δ_H_ 0.88 (H-21), a secondary methyl group. In the up-field area, the δ_H_ 0.33 and 0.55 peaks appeared as a pair of doublets, suggesting the existence of the cycloartane cyclopropane structure. Two signals at δ_H_ 3.27 (H-3) and 3.31 (H-24) reveal the presence of carbinolic protons related to the hydroxyl groups attached. The two carbinolic carbon shifts at δ_C_ 78.99 (C-3) and 79.77 (C-24) revealed the presence of two secondary hydroxyl groups and a tertiary hydroxyl group from a hydroxylated quaternary carbon at δ_C_ 73.36 (C-25).

Compound **9** showed the molecular formula of C_29_H_48_O_2_. From the comparison of the spectroscopic data with other similar compounds published in the literature, it was determined to be 7α-hydroxystigmasterol [28,29]. The ^1^H NMR showed three secondary methyls at δ_H_ 0.93 (H-21), 0.81 (H-26), and 0.93 (H-27), two singlet methyls at δ_H_ 0.68 (H-18), and 0.99 (H-19), and one triplet signal at δ_H_ 0.83 (H-29) indicating a tertiary methyl. The olefinic proton at δ_H_ 5.64 (H-6) in down-fields correlated to the double bond between C5 and C6. Another doublet peak at δ_H_ 5.16 (H-22) in the same area suggests another double bond between C22 and C23. The shifted signal at δ_C_ 146.39 (C-5) from the ^13^C NMR spectra is a reference to the α-orientation of the hydroxyl group on C27.

## 4. Discussion

A great deal of ethnopharmacological and ethnomedicinal uses of natural terpenes from different plant resources are related to the presence of isoprenoid units. Recent scientific findings have demonstrated the important role of triterpenes in these therapeutic effects, especially anti-tumor, anti-inflammatory, and immunoregulatory aspects [9].

The rich terpenoid profile of the Iranian *Euphorbia* species has motivated researchers to carry out further investigations on the biological and pharmacological potentials of these species. As a result, many therapeutic effects are introduced for them together with *Euphorbia turcomanica,* which is proven to have anti-cancer and immunomodulatory functions [30]. On the other hand, these studies are mainly focused on the medicinal properties of whole or partial plant extracts and cannot specifically show which bioactive component is responsible for the majority of these effects. Hence it is important to carry out phytochemical studies as well to clear the ambiguity and identify each present compound along with its roles individually. This type of study will also be beneficial in order to recognize natural sources of bioactive compounds for drug development. 

The present study exhibits that the triterpenoid profile of *E. turcomanica* is not only remarkable, but also there is a vast variety of them with different subclasses, some of which are even considered to be scarce. 

Cycloartanes are triterpenoids identified by their tetracyclic skeletal framework, cyclopropane ring, and side chain. This class of triterpenoids is responsible for a range of biological activities and is considered one of the leading chemical compounds in *Euphorbia* species, being involved in the biosynthesis of sterols and, therefore, could be considered one of the distinctive chemotaxonomic biomarkers in this genus [9]. Three cycloartane-type triterpenes (**4**, **6**, **8**) are elucidated for the first time from *E. turcomanica*. Compounds **4** and **6** have been isolated from many members of Euphorbiaceae so far studied. They are also found simultaneously in species such as *E. schimperi* [31] and *E. altotibetic* [32]. Also, both of these compounds were isolated from *E. segetalis* and have shown antiviral activities against the African swine fever virus and Herpes simplex virus [33]. Compound **4** has shown notable cancer chemopreventive effects [34], and compound **6**, which has also been isolated from *E. spinidens* [25], is connected to a range of pharmacological effects such as anti-inflammatory, anti-diabetic and antioxidant activities which can lead to further therapeutic properties [35,36].

Compound **8** is a less common cycloartane in comparison, but still, it has been reported from *E. marschalliana* [37] and *E.denticulata,* which has shown potential cytotoxic activities [38].

Compound **3,** identified as isomultiflorenol, is another triterpene isolated from *E. turcomanica* during the present research, which has already been elucidated from other *Euphorbia* plants such as *E. pubescens* [18] and *E. supina* [39]. During one research experiment on isomultiflorenol, significant anti-cancer activities against human cervical cancer cells were shown [40], which makes this compound, and thus its resources, highly valuable in chemotherapy-related drug development.

Although the biological effects from the triterpene-containing extracts are mostly associated with cycloartanes, there are also other types of triterpenes identified during this research that are noteworthy due to their scarcity in *Euphorbia* species. Compound **7** is a known pentacyclic ursane-type triterpene that was previously isolated from other plant families [26], but to our knowledge, there are no reports to show its presence in the species of Euphorbiaceae. Not enough studies have been carried out regarding this triterpene, and further investigations can be performed to reveal the potential effects of this natural compound.

Compound **2** is another compound isolated from *E. turcomanica* during the present research. It is a hopane-type triterpene which is considered one of the rare classes of triterpenes. The uncommon presence of a double bond between C-5 and C-6 causes the rare structural characterization of this compound. This compound has also been found in other *Euphorbia* species, including *E. lathyris* [41], and *E. aphylla* [42]. Moreover, previous studies have shown that this natural compound is associated with the anti-leishmanial activities of *E. peplus* [17]. Several studies have demonstrated the antimicrobial and antimycobacterial activities, respectively, from *E. neriifolia* and *Cissampelos mucronate* containing simiarenol as one of the present compounds in their extracts. Also, one recent research has shown this plant’s probable efficacy in treating a range of infectious diseases such as COVID-19, which might be related to this natural constituent but further investigations should be carried out to determine these claims and specifically relates them to simiarenol [43,44].

Two steroids (**5**, **9**) were also elucidated during the current research. Compound **5** is a commonly occurring phytosterol called β-sitosterol, which is a key component present in plants. Out of the many pharmacological and biological effects that have been reported for this compound over the past decades, anti-inflammatory, angiogenic, anti-adipogenic, anti-diabetic, anti-oxidant, anti-cancer, immunomodulatory activities and a role in cardiovascular diseases prevention can be mentioned, reported from recent findings that can be beneficial in drug developments and other therapeutic applications [45,46,47,48,49,50,51]. The hypocholesterolemic effects of *E. hirta* and its role in curing obesity have been linked to this natural compound [22], as well as the larvicidal activity of *E. thymifolia* [21]. β-sitosterol is also responsible for the antimicrobial activity of *E. segetalis* in the presence of other antimicrobial triterpenes [3]. In contrast with the frequency and availability of β-sitosterol in different species, the other isolated steroid (**9**) has not yet been reported from any of the *Euphorbia* species. Although this oxidized sterol has been isolated from other plant species [52], not many researchers have focused on its bioactive and pharmacological effects up until now.

One monoterpene (**1**) is also isolated during this study and identified as loliolide, which is a monoterpenoid lactone with 11 carbons and one of the degradation products of carotenoids that have been isolated from several higher plants and marine algae. Studies have revealed anti-bacterial and anti-fungal properties [53] as well as anti-oxidant [54,55], anti-inflammatory [56,57], potential anti-diabetic [58], and neuromodulatory effects [59,60]. Many studies on plant extracts from other families containing loliolide have been carried out that proved a wide range of anti-cancer activities on different cell lines [61,62,63,64,65,66,67]. So far, loliolide has been isolated from other species of the *Euphorbia* genus, such as *E. supina* [68], *E. micractina* [69], *E. cooperi* [70], and *E. alatavica* [71] but has never been reported to be identified in *E. turcomanica* before.

## 5. Conclusions

In the present study, nine known terpenoids were isolated from *E. turcomanica* for the first time, including an iridoid monoterpene, as well as steroids and triterpenes. The results suggest that this plant comprises a variety of compounds that can be considered a potential source of natural bioactive constituents with applications in medical and pharmacological sciences. Further investigation should be done for the isolation and identification of other phytochemical compounds in the *Euphorbia* genus, and it is worth exploring their prospective applications.

## Data Availability

The data presented in this study are available in the article.

## References

[1] Vasas A., Hohmann J. (2014). *Euphorbia* diterpenes: Isolation, structure, biological activity, and synthesis (2008–2012). Chem. Rev..

[2] Jassbi A.R. (2006). Chemistry and biological activity of secondary metabolites in *Euphorbia* from Iran. Phytochemistry.

[3] Pahlevani A.H., Liede-Schumann S., Akhani H. (2020). Diversity, distribution, endemism and conservation status of *Euphorbia* (Euphorbiaceae) in SW Asia and adjacent countries. Plant Syst. Evol..

[4] Pahlevani A.H., Riina R. (2011). A synopsis of *Euphorbia* subgen. *Chamaesyce* (Euphorbiaceae) in Iran. Ann. Bot. Fenn..

[5] Masyita A., Sari R.M., Astuti A.D., Yasir B., Rumata N.R., Emran T.B., Nainu F., Simal-Gandara J. (2022). Terpenes and terpenoids as main bioactive compounds of essential oils, their roles in human health and potential application as natural food preservatives. Food Chem. X.

[6] Shi Q.-W., Su X.-H., Kiyota H. (2008). Chemical and pharmacological research of the plants in genus *Euphorbia*. Chem. Rev..

[7] Kemboi D., Siwe-Noundou X., Krause R.W., Langat M.K., Tembu V.J. (2021). *Euphorbia* Diterpenes: An Update of Isolation, Structure, Pharmacological Activities and Structure–Activity Relationship. Molecules.

[8] Mitu S.A., Stewart P., Tran T.D., Reddell P.W., Cummins S.F., Ogbourne S.M. (2022). Identification of Gene Biomarkers for Tigilanol Tiglate Content in *Fontainea picrosperma*. Molecules.

[9] Kemboi D., Peter X., Langat M., Tembu J. (2020). A review of the ethnomedicinal uses, biological activities, and triterpenoids of *Euphorbia* species. Molecules.

[10] Saleem H., Ahmad I., Gill M.S.A. (2015). Phytochemical screening and diuretic activity of *Euphorbia granulata*. Bangladesh J. Pharm..

[11] Dey P.M., Harborne J.B. (1997). Plant biochemistry.

[12] Zare H., Noori A., Yusefzadi M., Banaee M. (2015). Acute toxicity of *Euphorbia turcomanica* on *Aphanius dispar*. Int. J. Aquat. Biol..

[13] Zolfaghari B., Yazdiniapour Z., Ghanadian M., Lanzotti V. (2016). Cyclomyrsinane and premyrsinane diterpenes from *Euphorbia sogdiana* Popov. Tetrahedron.

[14] Sukor S., Zahari Z., Rahim N., Yusoff J., Salim F. (2022). Chemical Constituents and Antiproliferative Activity of *Eleusine indica* (L.) Gaertn. Sains Malays..

[15] Yuan Z., Zheng X., Zhao Y., Liu Y., Zhou S., Wei C., Hu Y., Shao H. (2018). Phytotoxic compounds isolated from leaves of the invasive weed *Xanthium spinosum*. Molecules.

[16] Le D.K., Hoang M.H. (2020). Triterpenoids isolated from *Helicteres hirsuta*. J. Tech. Educ..

[17] Amin E., Moawad A., Hassan H. (2017). Biologically-guided isolation of leishmanicidal secondary metabolites from *Euphorbia peplus* L. Saudi Pharm. J..

[18] Carréu J.P.M. (2020). Bioactive Terpenoids from Euphorbia Pubescens: Isolation and Derivatization. Master’s Thesis.

[19] Ayatollahi A.M., Ghanadian M., Afsharypuor S., Mesaik M.A., Abdalla O.M., Shahlaei M., Farzandi G., Mostafavi H. (2011). Cycloartanes from *Euphorbia aellenii* Rech. f. and their Antiproliferative Activity. Iran J. Pharm Res..

[20] Takahashi S., Satoh H., Hongo Y., Koshino H. (2007). Structural Revision of Terpenoids with a (3 Z)-2-Methyl-3-penten-2-ol Moiety by the Synthesis of (23 E)-and (23 Z)-Cycloart-23-ene-3β, 25-diols. J. Org. Chem. Res..

[21] Rauter A.P., Filipe M.M., Prata C., Noronha J.P., Sampayo M.A., Justino J., Bermejo J. (2005). A new dihydroxysterol from the marine phytoplankton *Diacronema* sp. Fitoterapia.

[22] Ododo M.M., Choudhury M.K., Dekebo A.H. (2016). Structure elucidation of β-sitosterol with antibacterial activity from the root bark of *Malva parviflora*. Springerplus.

[23] Ghannadian M., Akhavan A., Abdalla O., Ayatollahi A., Mohammadi-Kamalabadi M., Ghazanfari H. (2013). Triterpenes from *Euphorbia spinidens* with immunomodulatory activity. Res. Pharm. Sci..

[24] Khan M.T.H., Khan S.B., Ather A. (2006). Tyrosinase inhibitory cycloartane type triterpenoids from the methanol extract of the whole plant of *Amberboa ramosa* Jafri and their structure–activity relationship. Bioorg. Med. Chem..

[25] Hajhashemi V., Ghanadian M., Palizaban A., Mahnam K., Eshaghi H., Gheisari B., Sadeghi-Aliabadi H. (2020). Cycloarta-23-ene-3beta, 25-diol a pentacyclic steroid from *Euphorbia spinidens*, as COX inhibitor with molecular docking, and in vivo study of its analgesic and anti-inflammatory activities in male swiss mice and wistar rats. Prostaglandins Other Lipid Mediat..

[26] Lima M., Braga P.A., Macedo M.L., Silva M., Ferreira A.G., Fernandes J.B., Vieira P.C. (2004). Phytochemistry of *Trattinnickia burserifolia*, *T. rhoifolia*, and *Dacryodes hopkinsii*: Chemosystematic implications. J. Braz. Chem. Soc..

[27] Ajithabai M., Sreedevi S., Jayakumar G., Nair M.S., Nair D.P., SP S.R. (2011). Phytochemical Analysis and Radical Scavenging Activity of the Extracts of *Costus picatus* Linn and *Coccinia indica* W and A, two Ethnic Medicinal Plants used in the Treatment of Diabetes mellitus. Free Radic. Antioxid..

[28] Johnsson L., Andersson R.E., Dutta P.C. (2003). Side-chain autoxidation of stigmasterol and analysis of a mixture of phytosterol oxidation products by chromatographic and spectroscopic methods. JAOCS J. Am. Oil Chem. Soc..

[29] Tasyriq M., Najmuldeen I.A., In L.L., Mohamad K., Awang K., Hasima N. (2012). 7α-Hydroxy-β-sitosterol from *Chisocheton tomentosus* induces apoptosis via dysregulation of cellular Bax/Bcl-2 ratio and cell cycle arrest by downregulating ERK1/2 activation. Evid. Based Complement. Altern. Med..

[30] Aliomrani M., Jafarian A., Zolfaghari B. (2017). Phytochemical screening and cytotoxic evaluation of *Euphorbia turcomanica* on Hela and HT-29 tumor cell lines. Adv. Biomed. Res..

[31] Abdel-Monem A.R., Abdel-Sattar E., Harraz F.M., Petereit F. (2008). Chemical Investigation of *Euphorbia schimperi* C. Presl. Rec. Nat. Prod..

[32] Li P., Feng Z.X., Ye D., Huan W., Da Gang W., Dong L.X. (2003). Chemical constituents from the whole plant of *Euphorbia altotibetic*. Helv. Chim. Acta.

[33] Madureira A., Ascenso J., Valdeira L., Duarte A., Frade J., Freitas G., Ferreira M. (2003). Evaluation of the antiviral and antimicrobial activities of triterpenes isolated from *Euphorbia segetalis*. Nat. Prod. Res..

[34] Kikuchi T., Akihisa T., Tokuda H., Ukiya M., Watanabe K., Nishino H. (2007). Cancer chemopreventive effects of cycloartane-type and related triterpenoids in in vitro and in vivo models. J. Nat. Prod..

[35] Badole S.L., Zanwar A.A., Khopade A.N., Bodhankar S.L. (2011). In vitro antioxidant and antimicrobial activity cycloart–23–ene–3β,-25–diol (B2) isolated from *Pongamia pinnata* (L. Pierre). Asian Pac. J. Trop. Med..

[36] Badole S.L., Mahamuni S.P., Bagul P.P., Khose R.D., Joshi A.C., Ghule A.E., Bodhankar S.L., Raut C.G., Khedkar V.M., Coutinho E.C. (2013). Cycloart-23-ene-3β, 25-diol stimulates GLP-1 (7–36) amide secretion in streptozotocin–nicotinamide induced diabetic Sprague Dawley rats: A mechanistic approach. Eur. J. Pharmacol..

[37] Jassbi A.R., Zamanizadehnajari S., Tahara S. (2004). Chemical constituents of *Euphorbia marschalliana* Boiss. Z Nat. C J. Biosci.

[38] Shamsabadipour S., Zarei S.M., Ghanadian M., Ayatollahi S.A., Rahimnejad M.R., Saeedi H., Aghaei M. (2018). A new taraxastane triterpene from *Euphorbia denticulata* with cytotoxic activity against prostate cancer cells. Iran. J. Pharm. Res..

[39] Tanaka R., Matsunaga S. (1991). Fernane and multiflorane triterpene ketols from *Euphorbia supina*. Phytochemistry.

[40] Li J., Chen Y. (2020). Anticancer activity of isomultiflorenol against human cervical cancer cells due to G2/M cell cycle arrest, autophagy and mitochondrial mediated apoptosis. Trop. J. Pharm. Res..

[41] Hemmers H., Gülz P.-G., Marner F.-J., Wray V. (1989). Pentacyclic triterpenoids in epicuticular waxes from *Euphorbia lathyris* L., Euphorbiaceae. Z. Für Nat. C.

[42] Gülz P.-G., Bodden J., Müller E., Marner F.-J. (1988). Epicuticular wax of *Euphorbia aphylla* brouss. ex. willd., Euphorbiaceae. Z. Für Nat. C.

[43] Sultana A., Hossain M.J., Kuddus M.R., Rashid M.A., Zahan M.S., Mitra S., Roy A., Alam S., Sarker M.M.R., Naina Mohamed I. (2022). Ethnobotanical Uses, Phytochemistry, toxicology, and pharmacological properties of *Euphorbia neriifolia* Linn. against infectious diseases: A comprehensive review. Molecules.

[44] Akande R., Fouche G., Famuyide I., Makhubu F., Nkadimeng S., Aro A., Kayoka-Kabongo P., McGaw L. (2022). Anthelmintic and antimycobacterial activity of fractions and compounds isolated from *Cissampelos mucronata*. J. Ethnopharmacol..

[45] Azemi A.K., Nordin M.L., Hambali K.A., Noralidin N.A., Mokhtar S.S., Rasool A.H.G. (2022). Phytochemical Contents and Pharmacological Potential of *Parkia speciosa* Hassk. for Diabetic Vasculopathy: A Review. Antioxidants.

[46] Karim S., Akhter M.H., Burzangi A.S., Alkreathy H., Alharthy B., Kotta S., Md S., Rashid M.A., Afzal O., Altamimi A.S. (2022). Phytosterol-Loaded Surface-Tailored Bioactive-Polymer Nanoparticles for Cancer Treatment: Optimization, In Vitro Cell Viability, Antioxidant Activity, and Stability Studies. Gels.

[47] Wang K.N., Hu Y., Han L.L., Zhao S.S., Song C., Sun S.W., Lv H.Y., Jiang N.N., Xv L.Z., Zhao Z.W. (2022). *Salvia chinensis* Benth Inhibits Triple-Negative Breast Cancer Progression by Inducing the DNA Damage Pathway. Front. Oncol..

[48] Elhady S.S., Ibrahim E.A., Goda M.S., Nafie M.S., Samir H., Diri R.M., Alahdal A.M., Thomford A.K., El Gindy A., Hadad G.M. (2022). GC-MS/MS Quantification of EGFR Inhibitors, β-Sitosterol, Betulinic Acid,(+) Eriodictyol,(+) Epipinoresinol, and Secoisolariciresinol, in Crude Extract and Ethyl Acetate Fraction of *Thonningia sanguinea*. Molecules.

[49] Shen C.-Y., Lee C.-F., Chou W.-T., Hwang J.-J., Tyan Y.-S., Chuang H.-Y. (2022). Liposomal β-Sitosterol Suppresses Metastasis of CT26/luc Colon Carcinoma via Inhibition of MMP-9 and Evoke of Immune System. Pharmaceutics.

[50] Vasanth K., Minakshi G.C., Velu K., Priya T., Kumar R.M., Kaliappan I., Dubey G.P. (2022). Anti-adipogenic β-sitosterol and lupeol from *Moringa oleifera* suppress adipocyte differentiation through regulation of cell cycle progression. J. Food Biochem..

[51] Witkowska A.M., Waśkiewicz A., Zujko M.E., Cicha-Mikołajczyk A., Mirończuk-Chodakowska I., Drygas W. (2022). Dietary plant sterols and phytosterol-enriched margarines and their relationship with cardiovascular disease among polish men and women: The WOBASZ II cross-sectional study. Nutrients.

[52] de Oliveira L.S., de Araújo M.F., Braz-Filho R., Vieira I.J.C. (2016). Dois Novos Diterpenos do Tipo Labdano e outros Compostos de *Conchocarpus cyrtanthus* (Rutaceae). Rev. Virtual Quim..

[53] Silva J., Alves C., Martins A., Susano P., Simões M., Guedes M., Rehfeldt S., Pinteus S., Gaspar H., Rodrigues A. (2021). Loliolide, a new therapeutic option for neurological diseases? In vitro neuroprotective and anti-inflammatory activities of a monoterpenoid lactone isolated from *Codium tomentosum*. Int. J. Mol. Sci..

[54] Radman S., Čižmek L., Babić S., Cikoš A.M., Čož-Rakovac R., Jokić S., Jerković I. (2022). Bioprospecting of less-polar fractions of *Ericaria crinita* and *Ericaria amentacea*: Developmental Toxicity and antioxidant activity. Mar. Drugs.

[55] Duan H., Wang G.C., Khan G.J., Su X.H., Guo S.L., Niu Y.M., Cao W.G., Wang W.T., Zhai K.F. (2021). Identification and characterization of potential antioxidant components in *Isodon amethystoides* (Benth.) Hara tea leaves by UPLC-LTQ-Orbitrap-MS. FCT.

[56] Chy M.N.U., Adnan M., Chowdhury M.R., Pagano E., Kamal A.M., Oh K.K., Cho D.H., Capasso R. (2021). Central and peripheral pain intervention by *Ophiorrhiza rugosa* leaves: Potential underlying mechanisms and insight into the role of pain modulators. J. Ethnopharmacol..

[57] Fernando I.P.S., Dias M.K.H.M., Madusanka D.M.D., Kim H.-S., Han E.-J., Kim M.-J., Seo M.-J., Ahn G. (2021). Effects of (–)-Loliolide against Fine Dust Preconditioned Keratinocyte Media-Induced Dermal Fibroblast Inflammation. Antioxidants.

[58] Lee D., Kim K.H., Jang T.S., Kang K.S. (2021). Identification of bioactive compounds from mulberry enhancing glucose-stimulated insulin secretion. Bioorganic Med. Chem. Lett..

[59] Sinan K.I., Chiavaroli A., Orlando G., Bene K., Zengin G., Cziáky Z., Jekő J., Fawzi Mahomoodally M., Picot-Allain M.C.N., Menghini L. (2020). Evaluation of pharmacological and phytochemical profiles of *Piptadeniastrum africanum* (Hook. f.) brenan stem bark extracts. Biomolecules.

[60] Jeyasri R., Muthuramalingam P., Suba V., Ramesh M., Chen J.-T. (2020). *Bacopa monnieri* and their bioactive compounds inferred multi-target treatment strategy for neurological diseases: A cheminformatics and system pharmacology approach. Biomolecules.

[61] Swantara M.D., Rita W.S., Dira M.A., Agustina K.K. (2022). Cervical anticancer activities of *Annona squamosa* Linn. leaf isolate. Vet. World.

[62] Gangadhar K.N., Rodrigues M.J., Pereira H., Gaspar H., Malcata F.X., Barreira L., Varela J. (2020). Anti-hepatocellular carcinoma (HepG2) activities of monoterpene hydroxy lactones isolated from the marine microalga *Tisochrysis lutea*. Mar. Drugs.

[63] Ahmed S.A., Rahman A.A., Elsayed K.N., Abd El-Mageed H., Mohamed H.S., Ahmed S.A. (2021). Cytotoxic activity, molecular docking, pharmacokinetic properties and quantum mechanics calculations of the brown macroalga *Cystoseira trinodis* compounds. J. Biomol. Struct. Dyn.

[64] Hamed A.N., Abouelela M.E., El Zowalaty A.E., Badr M.M., Abdelkader M.S. (2022). Chemical constituents from *Carica papaya* Linn. leaves as potential cytotoxic, EGFR wt and aromatase (CYP19A) inhibitors; a study supported by molecular docking. RSC Adv..

[65] El-Mekkawy S., Hassan A.Z., Abdelhafez M.A., Mahmoud K., Mahrous K.F., Meselhy M.R., Sendker J., Abdel-Sattar E. (2021). Cytotoxicity, genotoxicity, and gene expression changes induced by methanolic extract of *Moringa stenopetala* leaf with LC-qTOF-MS metabolic profile. Toxicon.

[66] Elasbali A.M., Al-Soud W.A., Al-Oanzi Z.H., Qanash H., Alharbi B., Binsaleh N.K., Alreshidi M., Patel M., Adnan M. (2022). Cytotoxic Activity, Cell Cycle Inhibition, and Apoptosis-Inducing Potential of *Athyrium hohenackerianum* (Lady Fern) with Its Phytochemical Profiling. Evid.-Based Complement. Altern. Med..

[67] Stojakowska A., Galanty A., Malarz J., Michalik M. (2019). Major terpenoids from *Telekia speciosa* flowers and their cytotoxic activity in vitro. Nat. Prod. Res..

[68] Tanaka R., Matsunaga S. (1989). Loliolide and olean-12-en-3β, 9α, 11α-triol from *Euphorbia supina*. Phytochemistry.

[69] Tao Y., Tian Y., Xu W., Guo Q., Shi J. (2016). Terpenoids from *Euphorbia micractina*. Acta Pharm. Sin..

[70] Hlengwa S.S. (2018). Isolation and Characterisation of Bioactive Compounds from Antidesma Venosum E. Mey. ex Tul. and *Euphorbia cooperi* NE Br. ex A. Berger. Master’s Thesis.

[71] Rozimamat R., Kehrimen N., Aisa H.A. (2019). New compound from *Euphorbia alatavica* Boiss. Nat. Prod. Res..

